# Interleukin-6 is elevated in synovial fluid of patients with focal cartilage defects and stimulates cartilage matrix production in an *in vitro *regeneration model

**DOI:** 10.1186/ar4107

**Published:** 2012-12-03

**Authors:** Anika I Tsuchida, Michiel Beekhuizen, Marijn Rutgers, Gerjo JVM van Osch, Joris EJ Bekkers, Arjan GJ Bot, Bernd Geurts, Wouter JA Dhert, Daniel BF Saris, Laura B Creemers

**Affiliations:** 1 Department of Orthopaedics, University Medical Center Utrecht, Heidelberglaan 100, Utrecht, 3584 CX, The Netherlands; 2 Department of Orthopaedics and Department of Otorhinolaryngology, Erasmus MC, University Medical Center Rotterdam, 's-Gravendijkwal 230, Rotterdam, 3015 CE, The Netherlands; 3 Faculty of Veterinary Medicine, University of Utrecht, Yalelaan 1, Utrecht, 3584 CL, The Netherlands; 4 Department of Tissue Regeneration, MIRA institute, University of Twente, Drienerlolaan 5, Enschede, 7522 NB, The Netherlands

## Abstract

**Introduction:**

This study aimed to determine whether, as in osteoarthritis, increased levels of interleukin-6 (IL-6) are present in the synovial fluid of patients with symptomatic cartilage defects and whether this IL-6 affects cartilage regeneration as well as the cartilage in the degenerated knee.

**Methods:**

IL-6 concentrations were determined by ELISA in synovial fluid and in conditioned media of chondrocytes regenerating cartilage. Chondrocytes were obtained from donors with symptomatic cartilage defects, healthy and osteoarthritic donors. The effect of IL-6 on cartilage regeneration and on metabolism of the resident cartilage in the knee was studied by both inhibition of endogenous IL-6 and addition of IL-6, in a regeneration model and in osteoarthritic explants in the presence of synovial fluid, respectively. Readout parameters were DNA and glycosaminoglycan (GAG) content and release. Differences between controls and IL-6 blocked or supplemented samples were determined by univariate analysis of variance using a randomized block design.

**Results:**

Synovial fluid of patients with symptomatic cartilage defects contained more IL-6 than synovial fluid of healthy donors (*P *= 0.001) and did not differ from osteoarthritic donors. IL-6 production of osteoarthritic chondrocytes during cartilage regeneration was higher than that of healthy and defect chondrocytes (*P *< 0.001). Adding IL-6 increased GAG production by healthy chondrocytes and decreased GAG release by osteoarthritic chondrocytes (*P *< 0.05). Inhibition of IL-6 present in osteoarthritic synovial fluid showed a trend towards decreased GAG content of the explants (*P *= 0.06).

**Conclusions:**

Our results support a modest anabolic role for IL-6 in cartilage matrix production. Targeting multiple cytokines, including IL-6, may be effective in improving cartilage repair in symptomatic cartilage defects and osteoarthritis.

## Introduction

Cytokines are thought to play an important role in articular cartilage degeneration [1]. In rheumatoid arthritis (RA), the pro-inflammatory cytokines tumor necrosis factor-α (TNF-α) and interleukin-1 (IL-1) are known to have pivotal roles in its pathophysiology [2]. In addition to IL-1 and TNF-α, interleukin-6 (IL-6) has been demonstrated to play a role in cartilage degeneration in RA. In mice models of RA, cartilage destruction was shown to be dependent on IL-6 [3,4]. Furthermore, tocilizumab, a humanized monoclonal antibody against the IL-6 receptor, now has an established role in the treatment of RA [5]. Besides efficacy in the amelioration of clinical signs and symptoms, tocilizumab has also been demonstrated to reduce joint space narrowing and levels of cartilage degradation biomarkers [6-8].

Although not as pronounced as in RA, mild and intermittent inflammation is frequently observed in symptomatic focal cartilage lesions, a condition thought to predispose to the development of osteoarthritis (OA), and in OA. Elevated concentrations of inflammatory mediators, including IL-6, have been found in the serum and synovial fluid of OA patients [9-16] and correlated to radiographic knee OA [17,18]. However, the presence of IL-6 in joints with symptomatic cartilage defects has not been evaluated until now. In other joint injuries known to predispose to OA, such as anterior cruciate ligament (ACL) injuries [19-21] and meniscal tears [12,22], increased levels of IL-6 have been detected in the synovial fluid. High levels of intra-articular inflammatory cytokines may, in addition to causing degeneration, also hamper tissue regeneration as cartilage repair is affected by the composition of the synovial fluid [23-25].

In OA most of the IL-6 present in the knee originates from the synovium [26]. However, chondrocytes in culture are capable of producing IL-6, albeit at low levels under most conditions [27-29]. Various stimuli, such as inflammatory molecules [30,31] and binding of (fragmented) matrix components, which bind through discoidin domain receptor 2 (DDR2) [32,33], have been reported to induce IL-6 synthesis, and these mechanisms are also proposed to play a role in OA. Chondrocytes can be stimulated by IL-6 either by binding directly to the gp80 receptor or, more commonly, through trans-signalling, in which IL-6 binds first to the soluble IL-6 receptor α (IL-6Rα) in the synovial fluid, and then forms a heterodimeric association with the membrane-bound gp130 receptor [34].

Despite its possible role in OA, studies investigating the role of IL-6 in OA models have provided inconsistent results. *In vitro *stimulation of chondrocytes with IL-6 has revealed anabolic effects, such as up-regulation of tissue inhibitor of metalloproteinases-1 (TIMP-1) [35] and type II collagen [36], as well as catabolic effects, such as down-regulation of cartilage matrix genes [37,38], inhibition of proteoglycan synthesis [39] and stimulation of aggrecanase production [40,41]. *In vivo *models have also revealed both chondroprotective and chondrodegenerative properties of IL-6. A protective role of IL-6 in a spontaneous OA model was reported in aging male mice [42], but through both mechanically induced OA and OA induced by hypoxia-inducible factor-2α (HIF-2α), IL-6 was identified as the mediator of cartilage degradation [43]. However, many other studies have failed to demonstrate a direct effect of IL-6 on cartilage matrix metabolism [44-46]. One of the explanations for this lack of effect may lie in the simplified set-up of many studies in which IL-6 has been added, since the action of IL-6 may depend on other factors in the joint, in particular in the synovial fluid.

This study evaluated the presence of IL-6 in the synovial fluid of patients with symptomatic cartilage lesions and patients with late stage OA, its production by chondrocytes isolated from these patients, and its role in cartilage regeneration. In addition, to evaluate the possible effects of high levels of IL-6 in the synovial fluid on cartilage in the knee, we cultured OA cartilage explants in the presence of OA synovial fluid in which IL-6 was selectively inhibited.

## Materials and methods

### Synovial fluid and cartilage sample collection and cell isolation

Collection of all patient material was done according to the Medical Ethical regulations of the University Medical Center Utrecht and according to the guideline 'good use of redundant tissue for clinical research' constructed by the Dutch Federation of Medical Research Societies on collection of redundant tissue for research [47]. This study does not meet the definition of human subjects' research or require informed consent and anonymous use of redundant tissue for research purposes is part of the standard treatment agreement with patients in our hospital [48].

Macroscopically healthy articular cartilage (*n *= 6) and synovial fluid (*n *= 20, age 25 to 47, average 40 years) were obtained from donors without any history of major joint trauma, osteoarthritis or inflammatory joint disease and absence of cartilage defects and synovial inflammation upon inspection of the knee within 24 hours post-mortem. Consistently, only one knee per donor was used. Defect cartilage (*n *= 3) and synovial fluid (*n *= 22, age 20 to 48, average 33 years) were obtained from donors undergoing either microfracture or autologous chondrocyte implantation (ACI) for focal grade III and IV cartilage defects (only grade III cartilage was used for chondrocyte isolation). During those procedures, the cartilage defect was debrided to remove all cartilage remnants down to the subchondral bone and create a stable cartilage rim. The debrided cartilage was used for chondrocyte isolation. Chondrocytes from this location were recently shown to have good regenerative capacities compared to cells harvested from non-weight bearing cartilage normally used for ACI [49]. Of the 22 patients with symptomatic cartilage defects, one had an associated ACL injury and a history of partial menisectomy, another three had previously received partial menisectomies and one had undergone an ACL reconstruction. OA cartilage (*n *= 12) and synovial fluid (*n *= 27, age 53 to 81, average 70 years) were obtained from donors undergoing total knee arthroplasty. Synovial fluid was centrifuged at 13,000 g for two minutes to remove debris, and stored at -80°C until use or analysis. Glucocorticoids affecting cytokine production are not prescribed at our institution for patients with focal cartilage lesions and patients with end-stage OA eligible for total knee replacement.

Cartilage samples were rinsed in phosphate buffered saline (PBS), cut into small pieces and enzymatically digested overnight at 37°C in a 0.15% collagenase type II (Worthington, Lakewood, NJ, USA) in Dulbecco's modified Eagle's medium (DMEM; Gibco, Life Technologies, Bleiswijk, The Netherlands) with penicillin/streptomycin (100 U/mL/100 μg/mL; Invitrogen, Life Technologies). After digestion, the cell suspension was filtered through a 70 μm cell strainer (BD Biosciences, San Diego, CA, USA), and the chondrocytes were spun down by 10 minutes centrifugation at 300 g.

### Measurement of IL-6 levels

To determine the IL-6 levels in the synovial fluids of healthy, defect and OA donors and in the conditioned media of healthy, defect and OA chondrocytes during regeneration, a multiplex ELISA was performed as previously described [50,51]. A total of 12 cytokines were measured of which IL-6 was most differentially regulated by healthy, defect and OA donors and hence chosen for further investigation. Briefly, specific antibodies (i.a. rat anti-human IL-6, MQ2-13A5; BD Biosciences) were coupled to carboxylated beads (Luminex Corporation, Austin, TX, USA). Recombinant human IL-6 (BD Biosciences, #550071) was used to make a standard curve. Synovial fluid samples were first treated with hyaluronidase (type IV-S, Sigma-Aldrich, Zwijndrecht, The Netherlands) at a concentration of 20 U/ml for 30 minutes at 37°C and then filtered by centrifuging through a polypropylene tube containing a 0.22 μm nylon membrane (Spin-X column; Corning, Amsterdam, The Netherlands). Subsequently, the synovial fluid samples were diluted 1:2 with HPE-0.1375% Tween (Sanquin, Amsterdam, The Netherlands). To block possible interfering antibodies present in the synovial fluid, the samples were diluted with an equal volume of 10% (v/v) normal rat and mouse serum (Rockland Immunochemicals Inc., Gilbertsville, PA, USA). Medium samples were directly incubated with the coupled beads. After incubation with the appropriate biotinylated antibodies (i.a. biotinylated rat anti-human IL-6, MQ2-39C3; BD Biosciences), samples were thoroughly washed and incubated with streptavidin-phycoerythrin (BD Biosciences) for 10 minutes. After washing, the samples were measured and analyzed using the Bio-Plex suspension system (Bio-Rad Laboratories, Hercules, CA, USA) with Bio-Plex Manager software, version 3.0. The concentration of IL-6 in the media and synovial fluid was expressed as pg/mL using the standard curves. Results of specific ELISAs for determination of IL-6 levels have previously been shown to be comparable to multiplex ELISA for conditioned medium, plasma and knee lavage samples [22,26,50].

### Regeneration culture

Isolated chondrocytes from healthy, defect and OA cartilage were expanded in a monolayer at 37°C and 5% CO_2 _at a seeding density of 5,000 cells per cm^2 ^in expansion medium consisting of DMEM, 10% fetal bovine serum (Hyclone, Thermo Scientific, Etten-Leur, The Netherlands), penicillin/streptomycin (100 U/mL/100 μg/mL) and 10 ng/mL basic fibroblast growth factor (bFGF; R&D Systems, Minneapolis, MN, USA). After two passages (P2), the chondrocytes were seeded on collagen type II-coated (chicken sternal cartilage; Sigma-Aldrich, #C9301) Millicell filters (Millipore Co., Bedford, MA, USA), at 1.6 × 10^6 ^cells per cm^2^. Chondrocytes were redifferentiated for 28 days in redifferentiation medium consisting of DMEM, 0.2 mM l-ascorbic acid-2-phosphate (AsAp; Sigma-Aldrich), 2% human serum albumin (Sanquin Blood Supply Foundation, Amsterdam, The Netherlands), penicillin/streptomycin (100 U/mL/100 μg/mL), 2% insuline-transferrine-selenium (ITS)-X (Invitrogen) and 5 ng/mL transforming growth factor-β2 (TGF-β2; R&D Systems). Since fibrillar type II collagen was previously shown to induce IL-6 release from chondrocytes [32,33], we also measured the release of IL-6 from P2 chondrocytes (*n *= 3 healthy donors) seeded at a density of 1.6 × 10^6 ^cells per cm^2 ^on filters coated with type I collagen (rat tail; BD Biosciences, #354249), and denatured type I and II collagen. Collagen was denatured by heating for 45 minutes at 70°C. Levels of IL-6 in the conditioned media were determined by specific ELISA for IL-6 (Cyto-set^®^; Invitrogen) according to the manufacturer's instructions.

Endogenous IL-6 production of defect chondrocytes proved to be not significantly different from healthy chondrocytes, which were both much lower than that of OA chondrocytes. Therefore, the role of IL-6 endogenously produced by defect chondrocytes (*n *= 3) and OA chondrocytes (*n *= 3) was studied through blockage of IL-6 with an activity-inhibiting antibody. To this end, the medium was supplemented with either 1 or 4 ug/mL anti-hIL-6 (purified mouse monoclonal IgG_1_; R&D Systems, #MAB206) or IgG_1 _isotype control (R&D Systems, #MAB002). The dose of anti-hIL-6 was chosen based on an IL-6-dependent murine plasmacytoma proliferation assay, as described earlier [52]. Furthermore, IL-6 activity was blocked with 100 ng/mL tocilizumab (RoActemra^®^, Roche, Woerden, The Netherlands), a humanized monoclonal antibody directed against the IL-6 receptor. The concentration of tocilizumab was chosen based on previously observed average concentrations of IL-6 receptor in the synovial fluid of patients with OA of approximately 10 to 40 ng/mL [15,16]. Since the effects of IL-6 inhibition were limited in expanded cells, we also verified the effects of IL-6 inhibition in freshly isolated OA cells (P0; *n *= 3).

Endogenous IL-6 production was relatively low in healthy chondrocytes, so the possible effects of high concentrations of IL-6 were further investigated by the addition of 10 ng/mL rhIL-6 with 25 ng/mL rhIL-6Rα (R&D Systems, #206-IL, #227-SR) to both healthy (*n *= 3) and OA (*n *= 3) chondrocytes. Medium was changed three times a week and supernatants were collected and stored at -80°C until later analysis. Per condition, six filters were seeded with chondrocytes, five for biochemical analyses and one for histological evaluation.

### Osteoarthritic cartilage explant culture

OA cartilage from three donors was cut into explants of approximately 1 mm by 1 mm with a mean wet weight (± SD) of 7.8 ± 2.8 mg. Cartilage explants were cultured for 14 days in explant medium consisting of DMEM, penicillin/streptomycin (100 U/mL/100 μg/mL), 1% ITS-X, 0.1 mM AsAp and 0.2% proline (Sigma-Aldrich), which was supplemented with either 0 or 25% (v/v) pooled OA synovial fluid from eight donors. Six explants per condition were used, five for biochemical analyses and one for histological evaluation. To study the role of IL-6 present in the synovial fluid, 4 ug/mL anti-hIL-6 or IgG_1 _isotype control and/or 100 ng/mL tocilizumab were added to the medium. Medium was changed three times per week and collected and stored at -20°C until later analysis.

The role of IL-6 was also studied by the addition of IL-6 to the culture medium. Explants from eight OA donors (11.3 ± 3.8 mg, minimum of three explants per condition) were pre-cultured for 24 hours in culture medium after which rhIL-6 (50 ng/mL) with IL-6 receptor (rhIL-6Rα; 200 ng/mL) [53] was added to experimental groups, but not to control groups. Explants were cultured for an additional 10 days with medium renewal every other day and the conditioned medium was collected and stored at -20°C until later analysis.

### Glycosaminoglycan and DNA analysis

After culture, the explants and the regenerated tissue were digested overnight in a papain buffer (250 μg/mL papain (Sigma-Aldrich) in 50 mM EDTA and 5 mM L-cysteine) at 56°C, followed by quantification of the glycosaminoglycans (GAGs) content using the dimethylmethylene blue (DMMB) assay [54]. The ratio of absorption at 540 nm to 595 nm was used to calculate the GAG content, using chondroitin-6-sulphate (shark; Sigma-Aldrich) as a standard. The GAG content in conditioned medium was also measured.

The DNA content in the papain digests was determined using a Picogreen DNA assay (Invitrogen) in accordance with the manufacturer's instructions.

### Histological evaluation

Both regenerated tissue and explants were fixed in 10% buffered formalin, dehydrated in alcohol, rinsed in xylene and infiltrated and embedded with paraffin. For histology, 5 μm sections were stained with safranin-O (Merck, Darmstadt, Germany) for GAG and counterstained with Weigert's haematoxylin (Klinipath, Duiven, The Netherlands) and 0.4% fast green (Merck) for nuclei and cytoplasm, respectively.

### Statistical analysis

All statistical analyses were performed using SPSS 18.0 (SPSS Inc., Chicago, IL, USA). Results are displayed as mean ± standard deviation (SD). Differences between controls and IL-6 blocked samples and differences between controls and IL-6 supplemented samples were determined by univariate analysis of variance using a randomized block design and *post hoc LSD*-test when four or more conditions were compared to each other. Differences in IL-6 concentration were determined by the Kruskal-Wallis test, using *post hoc *Mann-Whitney *U*-test and Bonferroni correction for synovial fluids, and using nested ANOVA with *post hoc t*-test and Bonferroni correction for conditioned media. Differences between the various collagen coatings were determined by univariate analysis of variance with *post hoc t*-test with Bonferroni correction.

## Results

### IL-6 in synovial fluid

**Figure 1:**
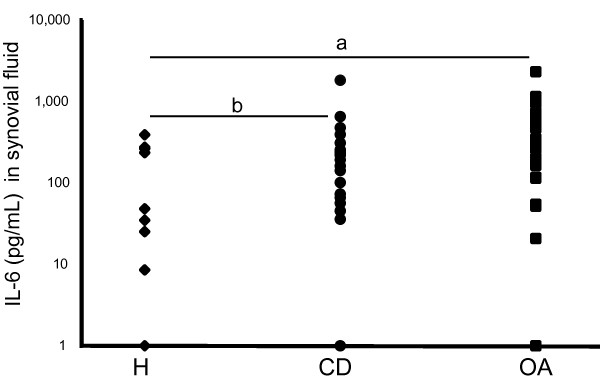
**Increased concentration of IL-6 in the synovial fluid of patients with cartilage damage**. Concentration of IL-6 in the synovial fluid of healthy (H), symptomatic cartilage defect (CD) and osteoarthritic (OA) donors. Note the logarithmic scale of the Y-axis; a = *P *< 0.001, b = *P *= 0.001.

The synovial fluid of donors with symptomatic cartilage lesions contained significantly more IL-6 than that of healthy donors (261 ± 385 pg/mL versus 64 ± 120 pg/mL, *P *= 0.001), and was slightly lower but not significantly different from OA patients (396 ± 508 pg/mL, Figure 1). IL-6 levels in the five patients with symptomatic focal cartilage defects and associated or previous ACL injury and/or partial menisectomy were not significantly different from the group as a whole (154 ± 70 pg/mL, *P *= 0.6).

### IL6 production in cell culture

**Figure 2:**
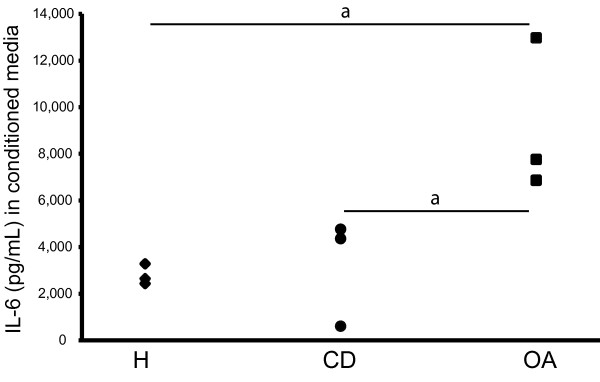
**Cartilage regeneration culture: IL-6 production**. Passage 2 expanded chondrocytes from healthy (H), defect (CD) and osteoarthritic (OA) cartilage were seeded at high density on type II collagen-coated filters and cultured during 28 days. Concentrations of IL-6 in conditioned media of chondrocytes during regeneration on Day 7 is shown; a = *P *< 0.001.

In regeneration cultures, chondrocytes of the various origins produced IL-6 reaching concentrations that were at least 10-fold higher than the concentrations present in the synovial fluid of the corresponding donor category (Figure 2). OA chondrocytes (9,368 ± 3,284 pg/mL) produced significantly more IL-6 than both healthy (2,814 ± 995 pg/mL) and defect chondrocytes (3,246 ± 2,089 pg/mL, *P *< 0.001). There was no significant difference in IL-6 production between healthy and defect chondrocytes.

To verify whether IL-6 production during regeneration was induced by the fibrillar type II collagen used for coating the filters in this model, we measured IL-6 production of regenerating chondrocytes on filters coated with various collagens. There was no difference in IL-6 production between type I and II collagen-coated filters and also not between native or denatured collagen-coated filters (Table 1). GAG and DNA content were also similar between the various coatings.

**Table 1:** Effect of collagen coating on IL-6 and cartilage matrix production

	Col I	Col II	Col ID	Col IID	*P*-value
IL-6 (pg/ml)	4,294 ± 2,152	4,604 ± 1,661	4,268 ± 1,446	5,463 ± 1,991	0.543
GAG (μg)	80 ± 24	85 ± 30	77 ± 25	85 ± 34	0.361
GAG release (μg/ml)	408 ± 29	410 ± 35	400 ± 38	498 ± 157	0.412
DNA (μg)	10 ± 1	9 ± 1	10 ± 2	10 ± 1	0.818

No significant differences were observed between type I collagen (Col I), type II collagen (Col II), denatured type I collagen (Col ID) and denatured type II collagen (Col IID) coating on IL-6 production at Day 7, GAG content after 28 days of culture, cumulative GAG release during 28 days of culture and DNA content after 28 days of culture. Results are displayed as mean ± SD.

### Regeneration culture

**Figure 3:**
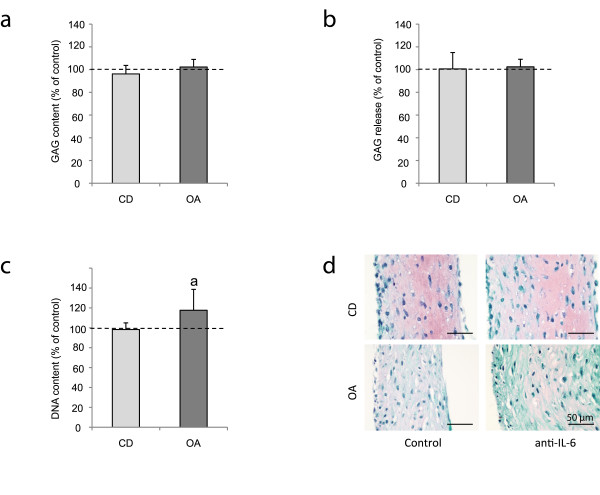
**Cartilage regeneration after inhibition of endogenously produced IL-6**. Cartilage regeneration cultures of three defect (CD) and three osteoarthritic (OA) donors with blockage of endogenous IL-6 with an activity-inhibiting antibody. (**a-c**) GAG content, GAG release and DNA content after 28 days of culture in IL-6 blocked samples depicted as percent of control samples (mean ± SD); a = *P *= 0.009. (**d**) Safranin-O staining of representative CD and OA chondrocyte donors regenerating either without (control; IgG isotype) or with inhibition (anti-IL-6) of endogenous IL-6. Scale bars indicate 50 μm.

To evaluate whether the high levels of IL-6 produced by the chondrocytes during regeneration play a direct role in cartilage regeneration, IL-6 was inhibited using an activity-inhibiting antibody during regeneration of P2-expanded defect- and OA chondrocytes. As no difference was found in IL-6 production between healthy and defect chondrocytes, only defect and osteoarthritic chondrocytes were studied. No effects were found on cartilage matrix production, although an increase in DNA content was found in OA chondrocytes (*P *= 0.009, Figure 3). Verification of these results using non-expanded osteoarthritic chondrocytes similarly showed no effect on cartilage matrix production and also the effect on DNA was no longer found (data not shown). Antagonism of the IL-6 receptor with tocilizumab in osteoarthritic chondrocytes failed to influence GAG and DNA content (data not shown).

**Figure 4:**
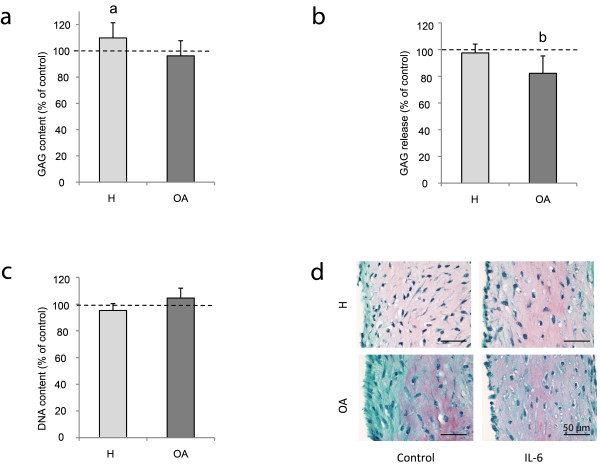
**Cartilage regeneration with addition of IL-6**. Cartilage regeneration cultures of three healthy (H) and three osteoarthritic (OA) donors with addition of rhIL-6 (10 ng/mL) and rhIL-6Rα (25 ng/mL). (**a-c**) GAG content, GAG release and DNA content of IL-6 supplemented samples depicted as percentage of control samples (mean ± SD); a = *P *= 0.009, b = *P *< 0.001. (**d**) Safranin-O staining of neocartilage generated by chondrocytes from healthy and osteoarthritic donors without or with addition of rhIL-6. Scale bars indicate 50 μm.

In healthy and defect chondrocytes endogenous IL-6 production was much lower than in OA chondrocytes. We, therefore, hypothesized that these cells could be more responsive to stimulation with exogenous IL-6 than OA chondrocytes. To examine whether exogenously added IL-6 could affect regeneration, 10 ng/mL rhIL-6 with 25 ng/mL rhIL-6Rα was added during regeneration culture of healthy and OA chondrocytes. In healthy chondrocytes, exogenous IL-6 increased GAG production in the neocartilage and a higher GAG/DNA ratio was found (*P *= 0.002, Figure 4A, D). In OA chondrocytes, IL-6 decreased GAG release (*P *< 0.001, Figure 4B) without affecting final GAG content in the neocartilage. DNA content was not modified by the addition of IL-6 (Figure 4C).

### Osteoarthritic explant culture

**Figure 5:**
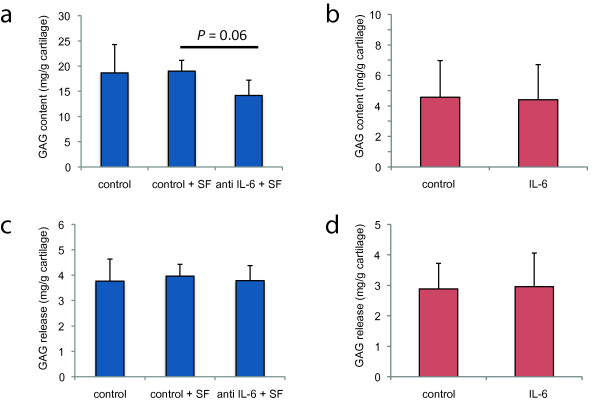
**OA cartilage explant culture with inhibition of synovial fluid IL-6 or addition of exogenous IL-6**. (**a, c**) GAG content and GAG release in OA cartilage explants from three donors cultured either in medium only, medium supplemented with 25% OA synovial fluid (SF) or medium with 25% OA synovial fluid in which IL-6 is blocked with an activity-inhibiting antibody (mean ± SD in mg/g); *P *= 0.06. (**b, d**) GAG content and GAG release in OA cartilage explants from eight donors cultured in the presence or absence of rhIL-6 (50 ng/mL) with rhIL-6Rα (200 ng/mL).

To study the effect of high levels of IL-6 present in the synovial fluid on resident cartilage in the knee, we performed OA cartilage explant studies in the presence of OA synovial fluid in which IL-6 was inhibited. Ideally, we would have also liked to perform these experiments using synovial fluid and cartilage explants from patients with chondral defects, but due to the very limited amount of material that can be obtained from these patients this was not feasible. In the absence of synovial fluid, inhibition of IL-6 did not alter the GAG and DNA content of the cartilage explants, nor was GAG release affected (data not shown). However, when IL-6 was inhibited in the presence of synovial fluid a trend towards a decreased GAG content of the explants was observed (*P *= 0.06, Figure 5A). In the absence of IL-6 inhibitors, the addition of synovial fluid increased the DNA content of explants (54.0 ± 28.0 μg/g cartilage versus 33.6 ± 20.9 μg/gr cartilage, *P *= 0.002), and this effect was abolished by blocking IL-6 (42 ± 20 versus 54 ± 28 μg/g cartilage). GAG release was neither affected by the addition of synovial fluid nor by inhibition of IL-6 (Figure 5C).

Exogenous IL-6 in combination with soluble IL-6 receptor in the absence of synovial fluid did not alter the GAG (Figure 5B) or DNA content of the explants and also did not modulate GAG release (Figure 5D).

## Discussion

In this study, we show increased IL-6 levels in the synovial fluid of patients with symptomatic cartilage defects compared to normal subjects. The IL-6 levels in patients with symptomatic cartilage defects were comparable to levels in patients with OA. Furthermore, we demonstrated for the first time that chondrocytes, especially OA chondrocytes, produce high concentrations of IL-6 during regeneration. Inhibition of this endogenously produced IL-6 did not affect cartilage matrix turnover, but addition of extra IL-6 increased the GAG content of neocartilage formed by healthy chondrocytes and decreased GAG release by osteoarthritic chondrocytes in an *in vitro *regeneration model. Furthermore, inhibition of IL-6 present in the synovial fluid showed a trend towards decreased matrix production in OA explants. Collectively, these results point towards an anabolic role of IL-6 in cartilage repair, albeit with limited effects.

Inflammatory mediators secreted by synovium and present in the synovial fluid have been demonstrated to affect cartilage regeneration *in vitro *[23-26,55]. Therefore, it is essential to characterize the mediators present in the synovial fluid of symptomatic cartilage defects and osteoarthritic joints and to determine their role in cartilage metabolism, in order to verify whether the outcomes of cartilage repair procedures, such as ACI, could potentially be enhanced by modulating the intra-articular environment. Levels of IL-6 comparable to those reported here were previously shown in the synovial fluid from healthy [10] and OA [10,12,56] joints; however, only limited data were available on IL-6 levels in joints with symptomatic focal cartilage defects. These are typically the joints that will be treated to stimulate regeneration of cartilage with techniques, such as ACI, and, therefore, of particular importance for regenerative medicine strategies. Only one study has reported on the levels of IL-6 in cartilage lesions of variable depth, but did not specify whether the damage was focal or whether more generalized OA-like cartilage degeneration was present in the knee [56], which is an important selection criterion for the indication of ACI. However, IL-6 levels seemed to correlate with the grade of cartilage damage as the synovial fluid concentration of IL-6 increased with lesion severity. In our study, only symptomatic focal grade III and IV cartilage lesions in otherwise healthy knees were included. This is more clinically relevant for cartilage regeneration, although no distinction was made between grade III and grade IV defects.

Most likely, IL-6 present in the synovial fluid originates from the cells in the synovial membrane [15]. In addition, adipose tissue, including that of the fat pad in the knee, is an important source of inflammatory mediators, including IL-6 [57,58], at least partly explaining the association of OA with obesity. Although we did not collect information regarding the body mass index (BMI) of the donors, it is possible that the OA donors were more obese, which could account at least partly for the higher levels of IL-6 found in OA synovial fluid. Furthermore, chondrocytes can produce IL-6, although chondrocytes embedded in their original matrix produce very little IL-6 [26]. However, during regeneration, chondrocytes produced high levels of IL-6, which, if they were then implanted intra-articularly, in theory, could cause high local concentrations and affect cartilage regeneration. The production of IL-6 was not a result of the type II collagen used in this model, as filters coated with type I collagen, which is not capable of inducing IL-6 [32], resulted in the same IL-6 production. Possibly, IL-6 production was induced by the TGF-β included in the current *in vitro *regeneration model, as was reported previously [29]. However, those data were obtained using non-expanded chondrocytes in the short term, rather than during regeneration culture. In addition, the differences in IL-6 production between various cell types cannot be explained by the addition of TGF-β, as this was the same for all cell types.

Little is known about the role of IL-6 during regeneration, but inhibition of IL-6 did not influence GAG or DNA content of the newly formed cartilage. Possibly the effect of IL-6 inhibition on cartilage formation was partly masked by the regenerative effect of TGF-β. However, addition of IL-6 did decrease GAG release by OA chondrocytes. In addition, in explant culture, no TGF-β was present and no clear effect was found here.

The results found here are partly in contrast to the previously described reduction of cartilage matrix gene expression and inhibition of proteoglycan synthesis [37-39,59]. Although the latter two effects of IL-6 can be seen as inhibitory, inhibition of synthesis can still be accompanied by a lack of net change in proteoglycan content if its deposition is increased. Indeed, we observed less GAG release into the medium without affecting final GAG content upon the addition of IL-6 to OA chondrocytes, suggesting that although total synthesis is reduced, final regeneration is the same. Unfortunately this aspect of cartilage regeneration is usually not addressed. Other differences between the current and previous studies evaluating the effect of IL-6 on chondrocyte metabolism may be based on the cells used, which were mainly bovine, porcine or rabbit chondrocytes. When human OA chondrocytes were used, actually no effect of IL-6 was seen on aggrecan production [44]. In the current study, addition of IL-6 to healthy chondrocytes, which produce much less IL-6, resulted in increased GAGs being deposited into the newly formed matrix. This is in line with previous studies using healthy human chondrocytes, revealing anabolic mechanisms upon addition of IL-6, such as up-regulation of TIMP-1 and bone morphogenetic protein-7 (BMP-7) [36,60]. Furthermore, in osteoarthritic explant culture, a trend towards decreased matrix production upon inhibition of IL-6 was observed. Exogenous IL-6 in combination with soluble IL-6 receptor in the absence of synovial fluid did not alter cartilage turnover, suggesting that IL-6 in the synovial fluid exerts its effects via interaction with other factors. The limited availability of synovial fluid restricted its presence in culture to 25% and, therefore, higher percentages may have yielded more pronounced effects. The limited effect of IL-6 inhibition on cartilage matrix turnover is unlikely to be due to a lack of inhibitory activity of the antibody, as this and other similar antibodies have demonstrated effectiveness in inhibiting IL-6 bioactivity in other models, including models with chondrocytes as target cells [39,61]. Diffusion limitations in the cartilage explants may have prevented complete penetration of the antibodies to inhibit locally produced IL-6, thus still allowing for paracrine signalling. However, this is probably limited as chondrocytes in their native extracellular matrix hardly produce IL-6 [26]. Penetration of tocilizumab, required for the inhibition of IL-6 signalling, into the newly formed cartilage during regeneration may have been suboptimal, allowing IL-6 signalling through the membrane-bound IL-6 receptor. This could explain the lack of effect that tocilizumab had during osteoarthritic chondrocyte regeneration.

The role of IL-6 in cartilage metabolism has been the subject of much debate. IL-6 is often described as a modulatory factor, because it can induce both anabolic and catabolic mechanisms. Recently, IL-6 injection into the knee joint of mice was described to cause cartilage destruction, but in that study, like in many others, supra physiological concentrations of IL-6 were used [43]. Concentrations of IL-6 similar to those found in the synovial fluid usually do not have effects on the expression of cartilage matrix proteins in cartilage [44-46]. To our knowledge, this is the first study to demonstrate an effect of physiological concentrations of IL-6 on cartilage matrix production during regeneration, albeit modestly. IL-6 is known to be induced by various catabolic stimuli present in OA, such as IL-1β [30], prostaglandin E_2 _(PGE_2_) [31], increased shear stress [62] and extracellular matrix components such as hyaluronan fragments [63] and matrillin-3 [32]. IL-6, in turn, is capable of inducing factors, such as metalloproteinases (MMPs) [64], TGFβ [65], vascular endothelial growth factor (VEGF) [66] and many others which are important for tissue remodelling. In bone IL-6 also induces remodelling through increased osteoclastogenesis [67], which is thought to be important in the observed inhibition of radiographic disease progression in RA patients treated with tocilizumab [6,68]. IL-6 has also been shown to have anabolic effects on cartilage, both indirectly through the up-regulation of factors, such as TIMP-1 [60], BMP-7 [36] and TGFβ [65], as well as directly through the up-regulation of cartilage matrix proteins [36]. In another study, injection of IL-6 into the joint cavity of mice stimulated proteoglycan synthesis in cartilage [69], while IL-6 knockout mice showed more extensive naturally occurring cartilage loss [42] and reduced proteoglycan synthesis [42,70]. The current study indicates that IL-6 has a mainly anabolic role in *in vitro *cartilage regeneration, although the effects are not strong, with increased GAG production in healthy chondrocytes and decreased GAG release in OA chondrocytes. Possibly the IL-6 in the synovial fluid of patients with symptomatic cartilage defects is induced in the course of regeneration and plays a role in tissue regeneration after cartilage damage.

Although cartilage pathology seemed to clearly affect IL-6 production and at least part of the response to interference with this factor, several other donor-related factors may have additionally influenced the results. To start with, there is the typical age difference found between OA donors (average 70 years [71]) and patients with cartilage defects, which usually present around 30 years of age [72]. However, IL-6 levels were not found to correlate with age [73]. The use of post-mortem collection of healthy synovial fluid and cells as opposed to the intra-operative collection of OA and cartilage defect synovial fluid and cells is less likely to have affected the results. Previous work from our group showed that there was no difference in viability between freshly isolated chondrocytes from healthy (post-mortem obtained) and grade III cartilage defect tissue [49], which is in line with the observation that viability and cartilage matrix content is very much unaltered within the first 24 hours [74-76]. Synovial fluid is contained in a relatively isolated compartment [77,78] and when kept at 4°C, levels of IL-6 have been shown to be stable for more than six hours [79].

## Conclusions

This study indicates that the level of IL-6 is increased in joints with symptomatic cartilage defects or OA compared to healthy joints. Moreover, the elevated levels of IL-6 appear to promote anabolic metabolism of the resident chondrocytes, and seem beneficial for formation of neocartilage during *in vitro *regeneration. Further research is necessary to evaluate whether targeting multiple cytokines or pathways, including IL-6, may be an effective means to improve cartilage matrix production during ACI or microfracturing in symptomatic cartilage defects or OA.

## Abbreviations

ACI: autologous chondrocyte implantation; ACL: anterior cruciate ligament; bFGF: basic fibroblast growth factor; BMI: body mass index; BMP-7: bone morphogenetic protein-7; DDR2: discoidin domain receptor 2; DMEM: Dulbecco's modified Eagle medium; DMMB: dimethylmethylene blue; GAG: glycosaminoglycan; HIF-2α: hypoxia-inducible factor-2α; IL-1: interleukin-1; IL-6: interleukin-6; IL-6Rα: interleukin-6 receptor α; MMP: metalloproteinase; OA: osteoarthritis; PBS: phosphate buffered saline; PGE_2_: prostaglandin E_2_; RA: rheumatoid arthritis; TIMP-1: tissue inhibitor of metalloproteinases 1; TGFβ: transforming growth factor β; TNF-α: tumor necrosis factor-α; VEGF: vascular growth factor

## Competing interests

The authors declare that they have no competing interests.

## Authors' contributions

AT, MB, MR, GvO, WD, DS and LC designed the study. JB and DS provided the study material. AT, MB, MR, JB, AB and BG acquired the data. AT, MB, MR, GvO, AB, BG, DS and LC analyzed and interpreted the data. AT and MB drafted the manuscript. MR, GvO, JB, AB, BG, WD, DS and LC critically revised the manuscript for important intellectual content. All authors read and approved the final manuscript.
